# Growth factors-based beneficial effects of platelet lysate on umbilical cord-derived stem cells and their synergistic use in osteoarthritis treatment

**DOI:** 10.1038/s41419-020-03045-0

**Published:** 2020-10-14

**Authors:** Li Yan, Li Zhou, Bo Yan, Lei Zhang, Wenxi Du, Fucun Liu, Qiang Yuan, Peijian Tong, Letian Shan, Thomas Efferth

**Affiliations:** 1 The First Affiliated Hospital, Zhejiang Chinese Medical University, Hangzhou, China; 2 Center for Stem Cell Translational Research, Zhejiang Chinese Medical University, Hangzhou, China; 3 Cell Resource Bank and Integrated Cell Preparation Center of Xiaoshan District, Hangzhou, China; 4 School of Biological and Chemical Engineering, Zhejiang University of Science and Technology, Hangzhou, China; 5 Department of Orthopaedics, Changzheng Hospital, Second Military Medical University, Shanghai, China; 6 College of Pharmacy, Zhejiang Chinese Medical University, Hangzhou, China; 7 Department of Pharmaceutical Biology, Institute of Pharmacy and Biochemistry, Johannes Gutenberg University, Mainz, Germany

**Keywords:** Stem cells, Mesenchymal stem cells

## Abstract

Poor viability of mesenchymal stem cells (MSCs) at the transplanted site often hinders the efficacy of MSCs-based therapy. Platelet lysate (PL) contains rich amounts of growth factors, which benefits cell growth. This study aimed to explore how human PL benefits umbilical cord-derived MSCs (huc-MSCs), and whether they have synergistic potential in osteoarthritis (OA) treatment. As quality control, flow cytometry and specific staining were performed to identify huc-MSCs, and ELISA was used to quantify growth factors in PL. CCK-8 and flow cytometry assays were performed to evaluate the effects of PL on the cell viability and cell cycle progression of huc-MSCs. Wound healing and transwell assays were conducted to assess the migration of huc-MSCs. RNA sequencing, real time PCR, and Western blot assays were conducted to explore the growth factors-based mechanism of PL. The in vitro results showed that PL significantly promoted the proliferation, cell cycle, and migration of huc-MSCs by upregulating relevant genes/proteins and activating beclin1-dependent autophagy via the AMPK/mTOR signaling pathway. The main growth factors (PDGF-AA, IGF-1, TGF-β, EGF, and FGF) contributed to the effects of PL in varying degrees. The in vivo data showed that combined PL and huc-MSCs exerted significant synergistic effect against OA. The overall study determined the beneficial effects and mechanism of PL on huc-MSCs and indicated PL as an adjuvant for huc-MSCs in treating OA. This is the first report on the growth factors-based mechanism of PL on huc-MSCs and their synergistic application. It provides novel knowledge of PLʹs roles and offers a promising strategy for stem cell-based OA therapy by combining PL and huc-MSCs.

## Introduction

Mesenchymal stem cells (MSCs) become an attractive and promising therapeutic tool for cell therapy and regenerative medicine, due to their self-renewal capacity, multilineage differentiation potential, paracrine effects, and immunomodulatory properties, which can promote all regenerative stages of damaged tissues. MSCs were firstly identified from bone marrow and thereafter were isolated from various tissues^1^. Bone marrow has been considered as the gold standard for MSCs isolation, but its acquisition is invasive and painful for donors^2^. Recently, new sources such as umbilical cord are preferred^2^. Umbilical cord is a noninvasive source with higher accessibility and fewer ethical constraints than bone marrow and other sources^3^. Human umbilical cord-derived MSCs (huc-MSCs) are noncontroversial compared to embryonic stem cells and induced pluripotent stem cells^2,4^. Moreover, huc-MSCs possess higher proliferative potential than MSCs from other sources^5^. Besides, huc-MSCs have an advantages of availability in large quantities, great expansion capability, no formation of teratomas or tumors, and strong immunomodulatory capacities^6^. It is known that MSCs can be self-protected from immunological defense during allogeneic transplantation, due to the expression of major histocompatibility complex (MHC) class I but not II^7^. Huc-MSCs are less immunogenic than other MSCs, making huc-MSCs a better candidate for allogeneic transplantation^3^. Currently, the FDA has registered dozens of clinical trials on the transplantation of huc-MSCs for treating refractory diseases, such as knee osteoarthritis (OA)^2,8^.

Under physiological conditions, platelets play an important roles not only in primary hemostasis but also in wound healing and tissue regeneration via the release of growth factors during activation. Abundant growth factors in platelet granules can be artificially obtained by freeze/thaw lysis, producing platelet lysates (PL). The primary factors in PL include platelet derived growth factor (PDGF), insulin-like growth factor (IGF), basic epidermal growth factor (EGF), fibroblast growth factor (FGF), and transforming growth factor-β (TGF-β), which can benefit cell proliferation, chemotaxis, and extracellular matrix production^9–11^. Most clinical applications of MSCs require sufficient *ex vivo* expansion to reach a meaningful cell number, and PL has a great potential to enable large-scale expansion of MSCs owing to these growth factors^12–14^. A positive dose-response relationship has been confirmed between platelet concentrations and MSCs proliferation^15^, indicating a correlation between PL and MSCs activities. Up to our knowledge, most studies have focused on the use of PL in MSCs culture as medium supplement to substitute fetal bovine serum (FBS), due to the safety concern of FBS about immune reactions and zoonotic infections^14^. The scientific rationale for the use of PL is the presence of the growth factors^16^. However, which factors contribute to the effects of PL and what mechanism they have, remains unknown.

A major technical impairment to MSCs-based cell therapy is the difficulty to isolate MSCs from tissue sources in which MSCs are present at low levels as well as the difficulty to culture these cells with sufficient quality and quantity. Successful isolation and rapid expansion of MSCs require a large amount of complete media containing bioactive supplement, such as FBS. Therefore, PL may not be suitable for MSCs culture as FBS substitute, because human blood is not usually available as routine source, although it gains advantages over FBS in safety. Given the rich growth factors in PL, we hypothesized that PL can benefit and strengthen MSCs in cell therapy as an adjuvant. To verify this hypothesis, we evaluated the multifaceted effects of PL on huc-MSCs at the cellular and molecular levels. Then, the roles of each growth factor in PL were explored. Given the known efficacy of MSCs on knee OA^17^, we employed a rat model of OA to evaluate the adjuvant role of PL in MSCs-based cell therapy. Recently, several reports have shown that PL can stimulate the proliferation of huc-MSCs and enhanced huc-MSCs-based bone tissue regeneration, but the underlying mechanism is unclear^18,19^. Also, the combination of PL and huc-MSCs for OA treatment has never been attempted. Therefore, this study would provide new information to the action mechanism of PL on MSCs and explore the synergistic application of combined PL and MSCs for OA therapy.

## Results

### Huc-MSCs identification

**Fig. 1:**
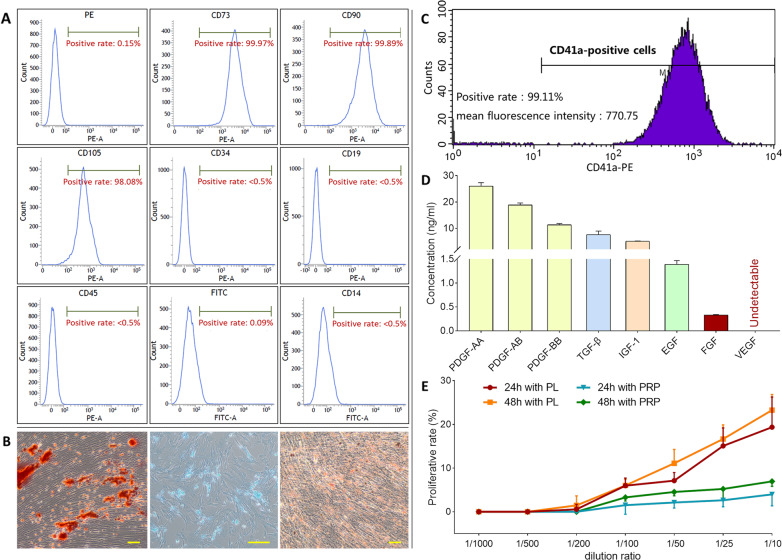
Identification of huc-MSCs and characterization of PL. The immunophenotype of huc-MSCs surface markers determined by flow cytometry (**A**). Specific staining of the cells after inducing three-line differentiation (scale bar = 100 μm): osteogenic differentiation (left) was examined by Alizarin red staining, chondrogenic differentiation (middle) was examined by Alcian blue staining, and adipogenic differentiation (right) was examined by Oil Red O staining (**B**). Flow cytometric pattern of CD41a expression on PRP (**C**). ELISA-tested concentration of PDGF-AA, PDGF-AB, PDGF-BB, TGF-β, IGF-1, EGF, FGF in PL (**D**). Cell viability of huc-MSCs upon treatment of PL and PRP for 24 and 48h (**E**). Data are mean values ± SD.

As shown in Fig. 1A, the umbilical cord-isolated cells showed fibroblast-like morphology with plastic adherent properties and expressed the surface markers CD73 (>99%), CD90 (>99%), and CD105 (>98%), but not CD14 (<0.5%), CD19 (<0.5%), CD34 (<0.5%), or CD45 (<0.5%), which complied with the international standard of MSCs^20^. Figure 1B showed that the cells have the ability of three-line differentiation (osteogenesis, chondrogenesis, and adipogenesis) when cultured in appropriate induction medium. The expression pattern and specific staining results indicated the cultured cells as typical MSCs.

### PL characterization

CD41a is a specific surface marker for human platelets. As shown in Fig. 1C, flow cytometrical analyses showed more than 99% positive expression of CD41a in PL production before freeze-thaw lysis. As shown in Fig. 1D, PL was obtained after the lysis, containing 26.25 ± 0.48 ng/ml PDGF-AA, 18.87 ± 0.74 ng/ml PDGF-AB, 11.31 ± 0.49 ng/ml PDGF-BB, 7.62 ± 1.29 ng/ml TGF-β, 5.05 ± 0.15 ng/ml IGF-1, 1.39 ± 0.08 ng/ml EGF, and 0.33 ± 0.01 ng/ml FGF.

### Proliferative effect of PL on huc-MSCs

The proliferative effects of PL and its precursor PRP on huc-MSCs were evaluated by CCK-8 assay. As shown in Fig. 1E, PL at a dilution rate range from 1/200 to 1/10 and PRP at a dilution rate range from 1/100 to 1/10 both exerted significant proliferative effects on huc-MSCs in a dose-dependent manner after 24 and 48 h treatment. The effect of PL at 1/50, 1/25, and 1/10 dilution rate was significant after 24 h and that at 1/100, 1/50, 1/25, and 1/10 dilution rate was significant after 48 h. The effect of PRP was only significant at dilution rates from 1/50 to 1/10 after 48 h treatment. Therefore, the effect of PL was better than that of PRP on huc-MSCs.

### RNAseq screening of molecular targets of PL on huc-MSCs

**Fig. 2:**
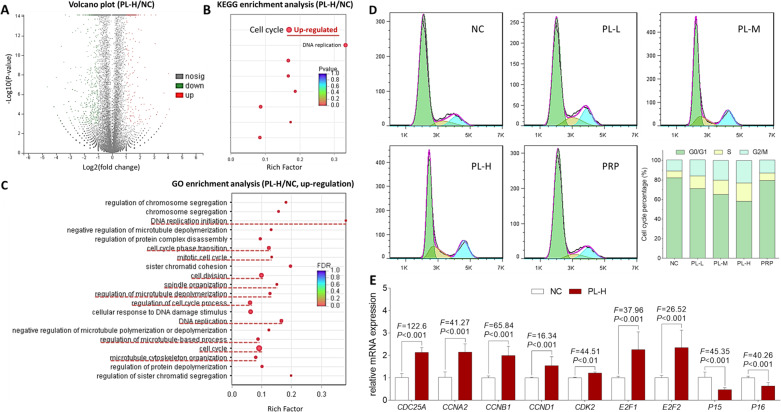
RNAseq and cell cycle analyses of PL on huc-MSCs. Volcano plot (**A**), KEGG analysis (**B**), and GO analysis (**C**) for the comparison between PL-H treated huc-MSCs and untreated huc-MSCs as assayed by RNAseq analysis. Cell cycle progression in huc-MSCs determined by flow cytometry (**D**), and the relative mRNA expressions of genes of interest in huc-MSCs detected by qPCR (**E**).

About 53–55 million reads with 80–83 hundred million bases of huc-MSCs were obtained. The error rate for each sample was less than 0.02%. More than 97.7% of total reads mapped to reference database genes. Statistical analysis revealed 774 differentially expressed genes (*P* < 0.01) out of 58,233 reference genes between PL-H-treated huc-MSCs and untreated huc-MSCs. The significantly upregulated and downregulated genes were functionally analyzed using GO and KEGG databases. As shown in Fig. 2B, the most significant and relevant pathways annotated by KEGG analysis were the cell cycle and cell proliferation (DNA replication) activated by PL. As shown in Fig. 2C, GO analysis showed a similar result that many of the PL-activated biological processes were closely related to the cell cycle and cell proliferation, such as DNA replication initiation, cell cycle phase transition, mitotic cell cycle, cell division, spindle organization, regulation of cell cycle process, DNA replication, and cell cycle. Moreover, cell migration-related processes, such as regulation of microtubule depolymerization, regulation of microtubule-based process, and microtubule cytoskeleton organization, were found to be activated by PL through the GO analysis. Therefore, the RNAseq analysis preliminarily revealed PL′s mechanism associated with the activation of cell cycle, cell proliferation, and cell migration of huc-MSCs. The representative huc-MSCs genes upregulated by PL were shown in Table 1.

**Table 1:** Representative genes regulated by PL in the cell cycle pathway.

Gene ID	Gene name	FC (PL-H/NC)	Log2FC(PL-H/NC)	*P*-value	Regulation
ENSG00000117399	CDC20	3.683	1.881	<0.001	Up
ENSG00000164045	CDC25A	2.452	1.294	<0.001	Up
ENSG00000101224	CDC25B	2.193	1.133	<0.001	Up
ENSG00000158402	CDC25C	2.646	1.404	<0.001	Up
ENSG00000093009	CDC45	2.626	1.393	<0.001	Up
ENSG00000094804	CDC6	2.086	1.061	<0.001	Up
ENSG00000145386	CCNA2	2.244	1.166	<0.001	Up
ENSG00000134057	CCNB1	2.428	1.28	<0.001	Up
ENSG00000101412	E2F1	3.681	1.88	<0.001	Up
ENSG00000007968	E2F2	2.704	1.435	<0.001	Up

### Effect and relevant molecular action of PL on cell cycle progression of huc-MSCs

According to the RNAseq result, cell cycle progression was the primary action, by which PL affects huc-MSCs. To verify this effect, flow cytometry was performed. As shown in Fig. 2D, PL induced cell cycle progression of huc-MSCs from *G*_0_/*G*_1_ phase to S and *G*_2_/M phases. With increasing doses of PL, the *G*_0_/*G*_1_ phase percentage was progressively decreased, and the S and *G*_2_/M phases were increased, indicating a dose-dependent manner of PL. PRP also induced the cell cycle progression, but its effect was much less than PL-L. To elucidate the molecular action of PL, mRNA expressions of the cell cycle-related genes were detected by real time PCR (qPCR) assay. As shown in Fig. 2E, PL-M significantly upregulated the expressions of *CDC25A*, *CCNA2*, *CCNB1*, *CCND1*, *CDK2*, *E2F1*, and *E2F2* and downregulated the expressions of *P15* and *P16* in huc-MSCs (each *P* < 0.01 vs. NC).

### Effect and relevant molecular action of PL on cell migration capacity of huc-MSCs

**Fig. 3:**
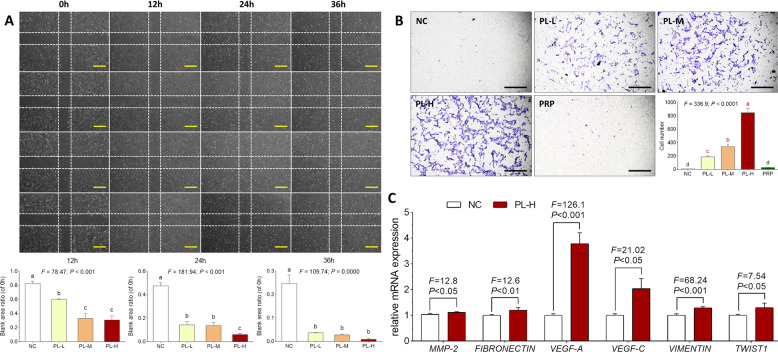
Observation and analysis of cell migrative effect of PL on huc-MSCs. Wounding healing assay of huc-MSCs with PL treatment at 24, 36, and 48h (**A**). Cell migration is represented as the ratio of the scratched wound area of huc-MSCs with 12, 24, and 36h treatment to the area without treatment (0h). Transwell migration assay of huc-MSCs with PL treatment (**B**), and the relative mRNA expressions of genes of interest in huc-MSCs detected by qPCR (**C**). Data are mean values ± SD. Different symbols (a, b, c, and d) indicate significant difference among groups [Fisher’s least significant difference (LSD), *P*<0.05], and the values decrease with the order from a to d.

Wound healing assay was conducted to evaluate the horizontal migration of huc-MSCs. As shown in Fig. 3A, the ratio of wound area with PL treatment at 12, 24, and 36 h to the area without treatment (0 h) was significantly decreased with elevated concentrations of PL (each *P* < 0.01 vs. NC), indicating that PL promoted wound closure of huc-MSCs by enhancing huc-MSCs migration in a dose-dependent manner. In particular, PL-H almost completed huc-MSCs wound closure after 36 h treatment. Transwell assay was conducted to detect the chemotactic migration of huc-MSCs. As shown in Fig. 3B, the migrated number of huc-MSCs to the bottom of chamber was significantly increased with the elevated concentrations of PL (each *P* < 0.01 vs. NC), indicating a dose-dependent manner of PL. However, there was no significant difference in migrated cell number between PRP-treated and untreated huc-MSCs. The result revealed that PL other than PRP raised huc-MSCs migration. qPCR assay was performed to detect mRNA expressions of the cell migration-related genes. As shown in Fig. 3C, PL-H significantly upregulated the expressions of *MMP2*, *FIBRONECTIN* (*FN1*), *VEGF-A*, *VEGF-C*, *VIMENTIN* (*VIM*), and *TWIST1* in huc-MSCs (each *P* < 0.01 vs. NC).

### Autophagy-related action mechanism of PL on huc-MSCs

**Fig. 4:**
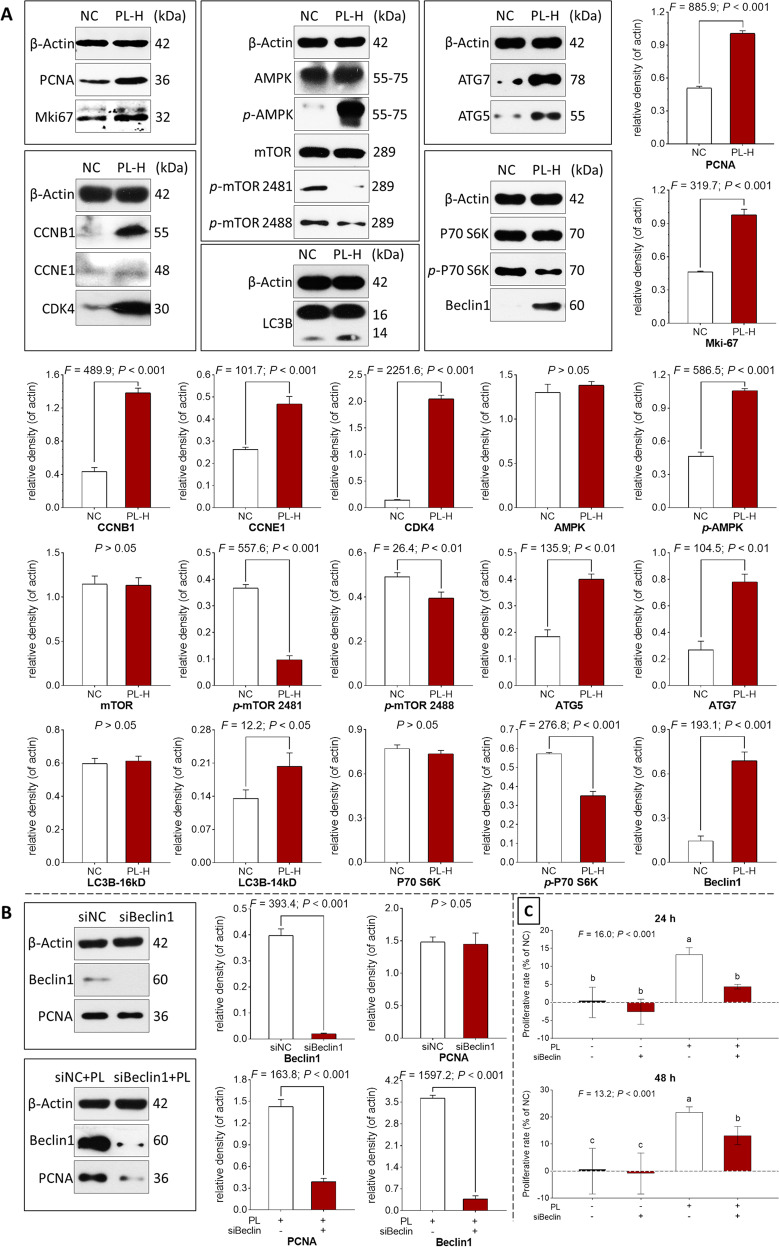
Western blot and cell viability analyses of huc-MSCs. Protein bands and protein expressions of huc-MSCs with PL treatment (**A**) and with PL plus *BECLIN1*-siRNA (siBeclin1) treatment (**B**). Cell viability of huc-MSCs with PL and siBeclin1 treatment at 24 and 48h (**C**). siNC: nontargeting control siRNA-treated group; siBeclin1: *BECN1*-siRNA-treated group. Data are mean values ± SD. Different symbols (a, b, and c) indicate significant difference among groups [Fisher’s least significant difference (LSD), *P* < 0.05], and the values decrease with the order from a to c.

Western blot (WB) assay was performed to explore the molecular actions of PL on cell cycle progression and cell proliferation of huc-MSCs. As shown in Fig. 4A, after 24 h treatment, PL significantly upregulated the protein expressions of cell proliferation (PCNA and Mki67) and cell cycle progression (CCNB1, CCNE1, and CDK4) (each *P* < 0.001 vs. NC). Moreover, PL significantly upregulated the expressions of phosphorylated AMPK (*p*-AMPK), phosphorylated mTOR (*p*-mTOR Ser2481/Ser2488), phosphorylated P79 S6K (*p*-P70 S6K), ATG5, ATG7, Beclin1, and LC3-II (each *P* < 0.001 vs. NC), indicating activation of the AMPK–mTOR–beclin1 signaling-related autophagy of huc-MSCs.

To verify the autophagy-related mechanism of PL, *BECN1*-siRNA was applied to block the autophagy of huc-MSCs by knocking down the beclin1 expression. As shown in Fig. 4B, *BECN1*-siRNA successfully blocked the beclin1 expression and significantly suppressed the expression of PCNA of huc-MSCs in the presence of PL treatment (each *P* < 0.001 vs. siNC). As shown in Fig. 4C, cell viability result showed that *BECN1*-siRNA significantly counteracted the PL-induced proliferation of huc-MSCs (*P* < 0.001 vs. siNC). These results verified that the beclin1-dependent autophagy participated in the proliferative mechanism of PL.

### Effect of PL-contained growth factors on huc-MSCs

**Fig. 5:**
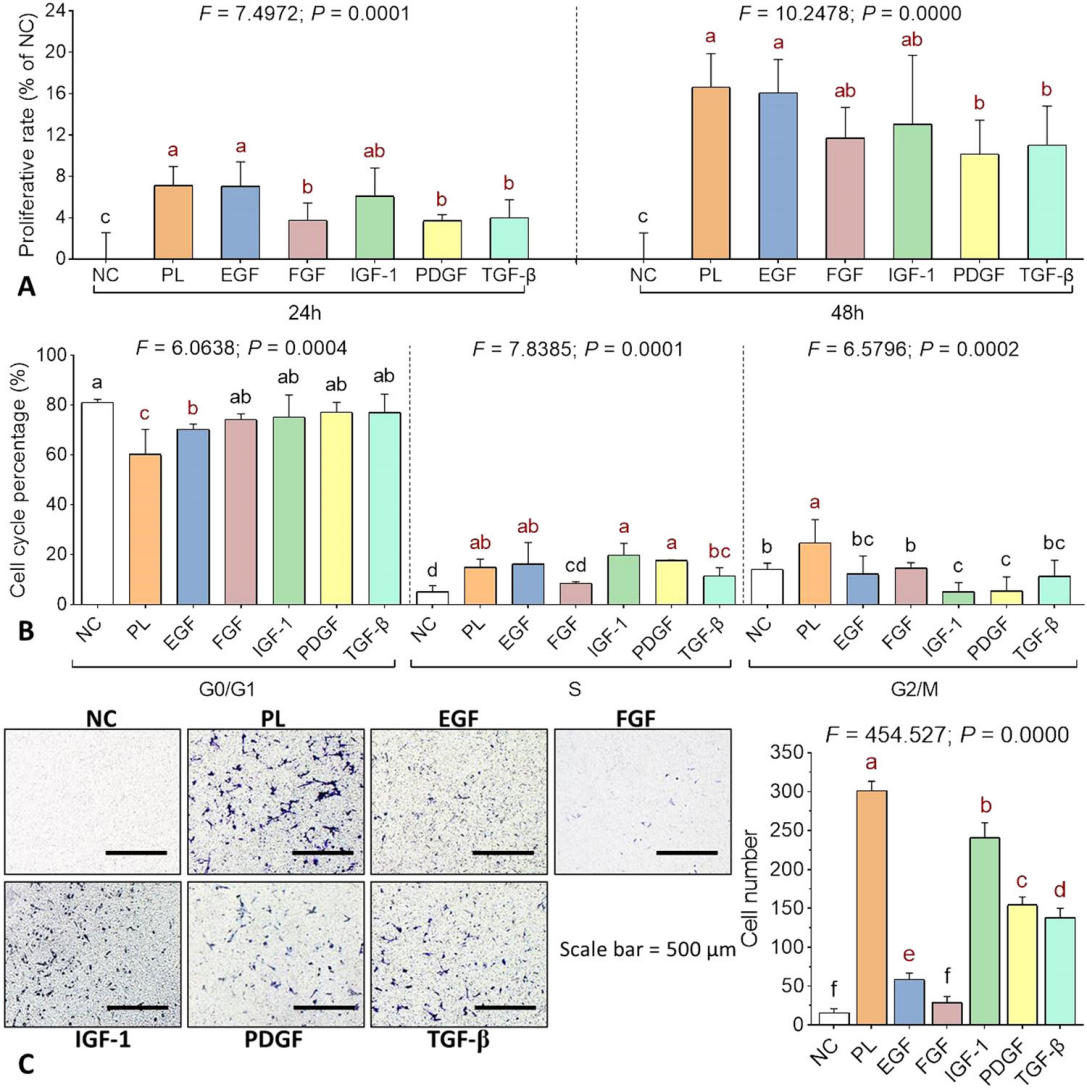
Individual effect of PL-contained growth factors on huc-MSCs. Cell viability (**A**), cell cycle (**B**) and cell migration (**C**) of huc-MSCs with treatment of PL and PL-contained growth factors. Data are mean values ± SD. Different symbols (a, ab, b, bc, c, cd, d, e, and f) indicate significant difference among groups [Fisher’s least significant difference (LSD), *P* < 0.05], and the values decrease with the order from a to f.

CCK-8 assay, cell cycle flow cytometry, and transwell assay were performed to evaluate the individual effects of each growth factor contained by PL. The concentrations of recombinant EGF (1.39 ng/ml), FGF (0.33 ng/ml), IGF-1 (5.05 ng/ml), PDGF (26.25 ng/ml), and TGF-β (7.62 ng/ml) were applied as same as those of the corresponding growth factors in PL. As shown in Fig. 5A, proliferate rate of huc-MSCs was significantly increased by PL and by each growth factor after 24 and 48 h treatment (each *P* < 0.05 vs. NC). Among those growth factors, EGF was most effective, followed by IGF-1 as the second most effective one, while FGF, PDGF, and TGF-β exerted lesser effects. The proliferative rate produced by EGF was approaching the highest level achieved by PL, indicating EGF as a main contributor to the proliferative effect of PL. As shown in Fig. 5B, cell cycle progression of huc-MSCs was significantly promoted by PL, EGF, IGF-1, PDGF, and TGF-β. EGF induced significant decrease of *G*_0_/*G*_1_ phase percentage and significant increase of S phase percentage as PL (each *P* < 0.05 vs. NC). FGF, IGF-1, PDGF, and TGF-β induced slight decreases of *G*_0_/*G*_1_ phase percentage (*P* > 0.05 vs. NC), followed by significant increases of S phase percentage induced by IGF-1, PDGF, and TGF-β (each *P* < 0.05 vs. NC). As shown in Fig. 5C, the migrated huc-MSCs number was significantly increased by PL, EGF, IGF-1, PDGF, and TGF-β (each *P* < 0.05 vs. NC). PL achieved the highest level, followed by IGF-1, PDGF, TGF-β, EGF, and FGF in the order of decreasing effects, indicating IGF-1 as a main contributor to the pro-migrative effect of PL.

### Molecular actions of PL-contained growth factors on huc-MSCs

**Fig. 6:**
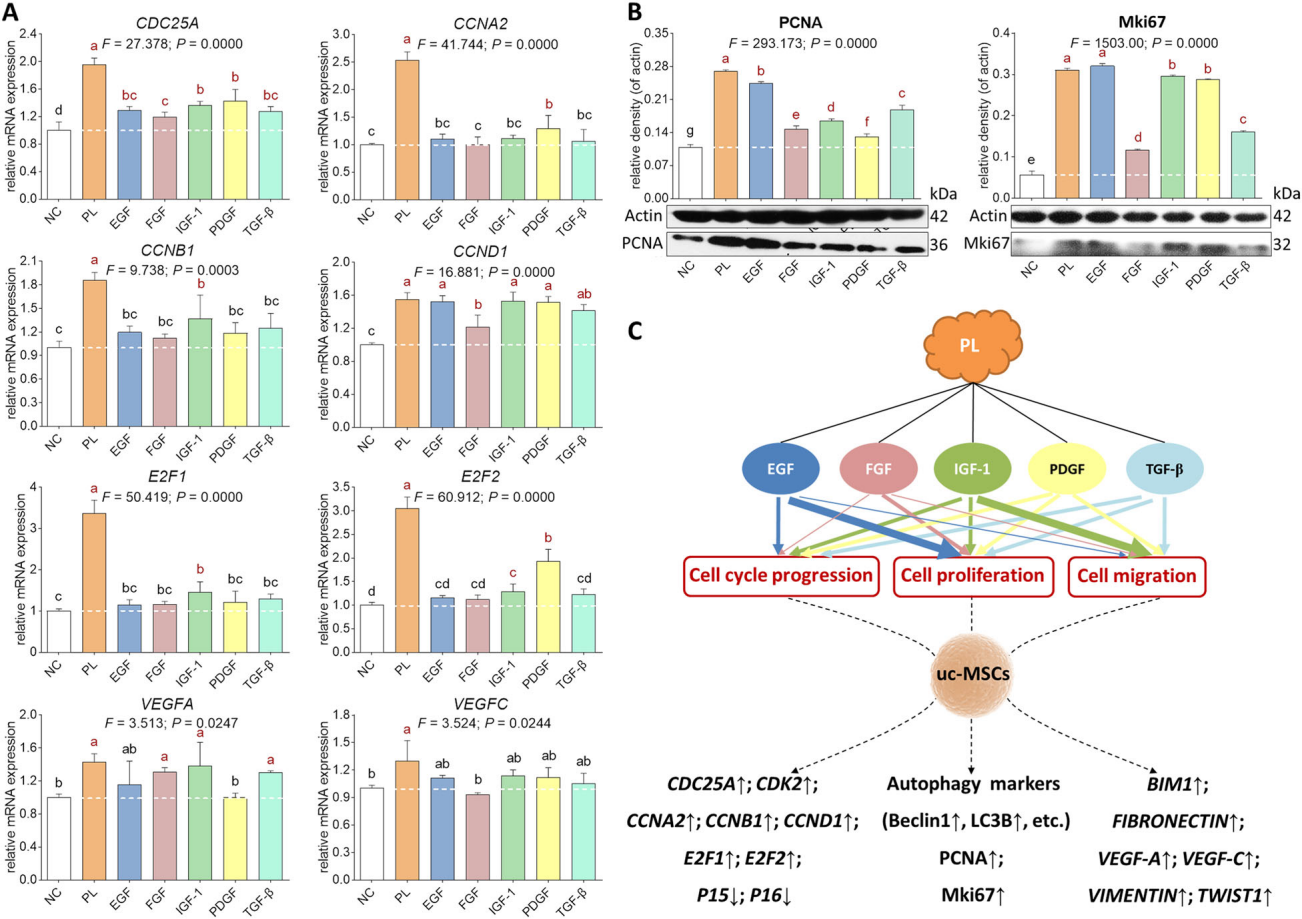
Molecular actions of PL-contained growth factors on huc-MSCs. qPCR analysis of mRNA expression (**A**) and Western blot analysis of protein expression (**B**) of huc-MSCs upon treatment of PL and PL-contained growth factors as well as a sketch diagram related to the effects and molecular actions of PL-contained growth factors (**C**). The thickness of size line positively represents the degree of effect of each growth factor. Data are mean values ± SD. Different symbols (a, ab, b, bc, c, cd, d, e, and f) indicate significant difference among groups [Fisher’s least significant difference (LSD), *P* < 0.05], and the values decrease with the order from a to f.

qPCR and WB assays were conducted to elucidate the PL- and growth factors-induced molecular changes in huc-MSCs. As shown in Fig. 6A, PL significantly up-regulated the mRNA expressions of *CDC25A*, *CCNA2*, *CCNB1*, *CCND1*, *E2F1*, *E2F2*, *VEGFA*, and *VEGFC*, while the growth factors performed various actions on these genes. *CDC25A* was significantly upregulated by PDGF, IGF-1, EGF, TGF-β, and FGF in a descending order (each *P* < 0.05 vs. NC). *CCNA2* was significantly upregulated by PDGF (*P* < 0.05 vs. NC) and statistically upregulated by IGF-1, EGF, and TGF-β in a descending order. *CCNB1* was significantly upregulated by IGF-1 (*P* < 0.05 vs. NC) and statistically upregulated by TGF-β, EGF, PDGF, and FGF in a descending order. *CCND1* was significantly upregulated by IGF-1, EGF, PDGF, TGF-β, and FGF in a descending order (each *P* < 0.05 vs. NC). *E2F1* was significantly upregulated by IGF-1 (*P* < 0.05 vs. NC) and statistically upregulated by TGF-β, PDGF, FGF, and EGF in a descending order. *E2F2* was significantly upregulated by IGF-1 and PDGF (each *P* < 0.05 vs. NC) and statistically upregulated by TGF-β, EGF, and FGF in a descending order. *VEGFA* was significantly upregulated by IGF-1, FGF, and TGF-β in a descending order (each *P* < 0.05 vs. NC) and statistically upregulated by EGF. *VEGFC* was statistically upregulated by IGF-1, PDGF, EGF, and TGF-β in a descending order. As shown in Fig. 6B, PL significantly upregulated protein expression of PCNA and Mki67, while the growth factors performed various actions on those proteins. The expression of PCNA was significantly increased by EGF, TGF-β, IGF-1, FGF, and PDGF in a descending order (each *P* < 0.05 vs. NC), and that of Mki67 was significantly increased by EGF, IGF-1, PDGF, TGF-β, and FGF in a descending order (each *P* < 0.05 vs. NC). The above data indicated that none of the growth factors exerted effects as strong as PL, but each one contributed to the effects of PL (Table 2).

**Table 2:** Summary of the effects of PL and growth factors on the cellular and molecular targets in huc-MSCs.

Targets		PL	EGF	FGF	IGF-1	PDGF	TGF-β
Cell viability		↑*	↑*	↑*	↑*	↑*	↑*
Cell cycle		↑*	↑*	↑	↑*	↑*	↑*
Cell migration		↑*	↑*	↑	↑*	↑*	↑*
*mRNA expression*							
*CDC25A*		↑*	↑*	↑*	↑*	↑*	↑*
*CCNA2*		↑*	↑	↑	↑	↑*	↑
*CCNB1*		↑*	↑	↑	↑*	↑	↑
*CCND1*		↑*	↑*	↑*	↑*	↑*	↑*
*E2F1*		↑*	↑	↑	↑*	↑	↑
*E2F2*		↑*	↑	↑	↑*	↑*	↑
*VEGFA*		↑*	↑	↑	↑*	↑*	↑*
*VEGFC*		↑*	↑	–	↑	↑	↑
*Protein expression*							
PCNA		↑*	↑*	↑*	↑*	↑*	↑*
Mki-67		↑*	↑*	↑*	↑*	↑*	↑*
Effective targets:		13	13	12	13	13	13
Significant targets		13	7	5	11	10	8

Positive effect with ↑; Significant effect with *; no effect with –.

### In vivo evaluation of adjuvant role of PL on huc-MSCs

**Fig. 7:**
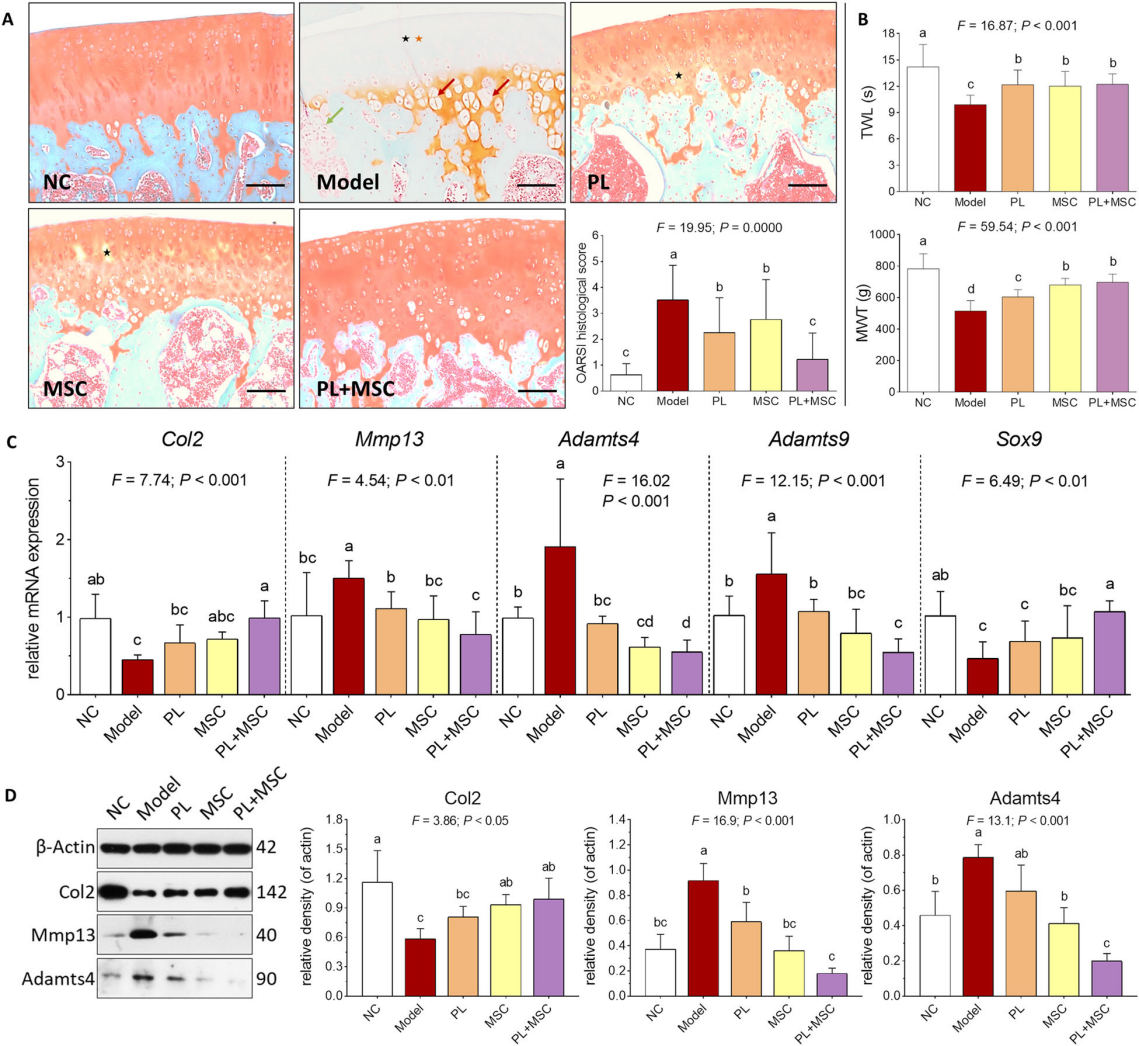
In vivo effect and mechanism of PL plus huc-MSCs on OA rats. Histopathological observation on cartilage (black asterisk: chondrocyte loss; orange asterisk: collagen disruption; red arrow: chondrocyte hypertrophy) and subchondral bone (green arrow: subchondral bone cyst) by using SO staining, and OARSI scoring of rat knee joints (**A**). Pain behavior observation (TWL and MWT) of rats (**B**). qPCR analysis of mRNA expression of OA pathological genes in cartilage tissue with treatment of PL, MSC, and PL+MSC (**C**). Western blot analysis of protein expression of OA pathological molecules in cartilage tissue with treatment of PL, MSC, and PL+MSC (**D**). Data are mean values ± SD. Different symbols (a, ab, b, bc, c, cd, and d) indicate significant difference among groups [Fisher’s least significant difference (LSD), *P* < 0.05], and the values decrease with the order from a to d. Scale bar = 100 μm.

According to the above results, PL improved cell proliferation, cell cycle transition, and cell migration of huc-MSCs through actions of PL-contained growth factors, indicating its adjuvant role on huc-MSCs. An OA rat model was employed to evaluate such adjuvant role in vivo. As shown in Fig. 7A, cartilage degeneration, characterized by chondrocyte hypertrophy and loss, collagen disruption, and subchondral bone cyst, was observed in the OA rat joints, while the degeneration was reversed, respectively by PL, MSC, and PL + MSC. With these treatments, the number of chondrocytes and mass of collagen were obviously increased, and the chondrocyte hypertrophy and subchondral bone cyst disappeared. Nevertheless, there remained several areas of slight collagen disruption in the PL-treated and MSC-treated samples, while no sign of degeneration was seen in the PL+MSC-treated samples. The OARSI scoring demonstrated that PL+MSC, MSC, and PL significantly decreased the histopathological score in a descending order (each *P* < 0.05 vs. model), indicating their anti-OA effects in the following order: PL+MSC > MSC > PL. TWL and MWT were classic pain behavior indices that reflect mechanical allodynia and thermal hyperalgesia respectively. As shown in Fig. 7B, TWL and MWT were significantly decreased with OA modeling (each *P* < 0.05 vs. NC) and significantly increased by PL+MSC, MSC, and PL (each *P* < 0.05 vs. Model). The effects of PL+MSC, MSC, and PL on TWL were similar to each other, and the effect of PL+MSC on MWT was similar to MSCs but better than PL. As shown in Fig. 7C, the mRNA expressions of *Col2* and *Sox9* were significantly downregulated and that of *Mmp13*, *Adamts4*, and *Adamts9* were significantly upregulated in the OA rat joints (each *P* < 0.01 vs. NC). These pathological changes were statistically reversed by PL, MSC, and PL+MSC in an order of increasing effects. PL+MSC exerted significant effects on all of these genes (each *P* < 0.01 vs. Model), while that of PL and MSC were significant only on *Mmp13*, *Adamts4*, and *Adamts9* (each *P* < 0.01 vs. Model). As shown in Fig. 7D, the protein expression pattern in joints was similar to the mRNA expression pattern that PL+MSC exerted the strongest reversal effects than that of PL or MSC on the abnormal expressions of Col2, Mmp13, and Adamts4 (each *P* < 0.01 vs. Model).

## Discussion

The main innovation of this paper is the finding of PL′s beneficial effects on huc-MSCs and its mechanism of growth factors-based action, which differs from previous studies focusing mainly on the potential of PL as substitute for FBS in MSCs expansion. For clinical applications, MSCs have to be expanded to reach a meaningful cell number, requiring a large amount of FBS-like supplement^13^. However, platelets lack *ex vivo* expanding capacity, resulting in unavailability of large-scale use of PL, especially for the autologous use. Thus, PL is more suitable for short-term use as an adjuvant for MSCs, but its adjuvant role has not yet been confirmed. A clinical study reported that the endogenous growth factors released from platelets in serum positively influenced the number of MSCs in vivo and induced proliferation of MSCs in vitro, and the effect had a direct relationship with platelets/growth factors^21^. Moreover, the growth factors of PL have been found to benefit the proliferation, chemotaxis, regeneration, and angiogenesis of MSCs^15^. Although the benefits of growth factors of PL to MSCs have been observed, the underlying mechanism remains poorly understood. To fill this gap, we conducted cellular and molecular experiments to investigate the adjuvant effect, and the growth factors-based mechanism of PL on huc-MSCs. Furthermore, an animal experiment was performed to determine the adjuvant role of PL in MSCs-based cell therapy. To our knowledge, this is the first study regarding this issue.

The platelet purity of our PL was above 99% before freeze-thaw lysis, and the contents of growth factors in PL were much higher than that released by PPR or other PL products^13^, making our product as growth factors-enriched PL (GF-PL). Cell viability assay showed that the GF-PL exerted stronger proliferative effect than PRP on huc-MSCs. To explore the mechanism of GF-PL, we conducted RNAseq analysis and found that GF-PL-targeted genes related to cell cycle progression, DNA replication, and microtubule-based activity of MSCs. Thus, we deduced that GF-PL exerted effects not only on proliferation, but also on cell cycle and migration of huc-MSCs. To verify this deduction, flow cytometry, wound healing, and transwell assays were conducted, and meanwhile, qPCR was employed to verify the RNAseq result. Our data demonstrated that GF-PL dose-dependently affected huc-MSCs by inducing cell cycle progression and migration. With these phenotypic changes, genes associated with cell cycle progression (*CDC25A*, *CCNA2*, *CCNB1*, *CCND1*, *CDK2*, *CDKN2A*, *CDKN2B*, *E2F1*, and *E2F2*) and cell migration (*MMP-2*, *FN1*, *VEGF-A*, *VEGF-C*, *VIM*, and *TWIST1*) as well as proteins associated with cell proliferation (PCNA and Mki67) and cell cycle progression (cyclin B, cyclin E, and CDK4) were all significantly regulated by GF-PL, indicating molecular actions of GF-PL on proliferation, cell cycle transition, and migration of huc-MSCs. It has been reported that self-renewal of human stem cells is controlled by the cell cycle regulatory machinery involving cyclins, CDKs, and E2F^22^, suggesting that GF-PL may enhance self-renewal ability of huc-MSCs. Moreover, the migration-related genes are known as metastatic genes of cancer cells, suggesting that GF-PL may have a metastasis-like promigrative mechanism, which confers strong mobility to huc-MSCs.

Autophagy is a mechanism essential for maintaining cellular characteristics and physiological functions of MSCs. Accumulating evidences have highlighted that autophagy serves to promote proliferation and inhibit apoptosis and inflammatory responses of MSCs^23,24^. And, activation of autophagy contributes to adaptive cell cycle response of MSCs^25^. Moreover, modulation of autophagy may have an impact on the regenerative and therapeutic potential of MSCs^26^. Beclin1 acts as a crucial modifier of autophagy involved in the activation of autophagy and formation of autophagosomes. In this study, canonical autophagy machinery (e.g., Beclin1, ATG5, ATG7, and LC3-II) of huc-MSCs was activated by GF-PL, and knock-down of beclin1 expression by *BECN1*-siRNA resulted in the suppression of GF-PL-induced proliferation and PCNA expression of huc-MSCs. Therefore, our data indicated an autophagy-mediated mechanism of GF-PL on the proliferation of MSCs. In future, further studies are needed to explore whether autophagy contributes to the mechanism of GF-PL on the cell cycle progression and migration of huc-MSCs and whether autophagy determines the therapeutic potential of huc-MSCs.

In GF-PL, the contents of growth factors were found in the following order: PDGF-AA > PDGF-AB > PDGF-BB > TGF-β > IGF-1 > EGF > FGF. Previous studies have demonstrated that each of these factors has positive effect on MSCs (Table 3)^27–61^. Of them, PDGF, IGF-1, and FGF promote proliferation and cell cycle transition of huc-MSCs, while the effects of TGF-β and EGF on huc-MSCs remain undetermined (Table 3)^30,39,41,54,58^. Moreover, no literature was found regarding the promigrative effect of any of these factors on huc-MSCs. In this study, we firstly compared the activities among the three forms of PDGF. As shown in Fig. S1, PDGF-AA, PDGF-AB, and PDGF-BB at 20 ng/ml induced proliferation and migration as well as PCNA expression of huc-MSCs in the following order of effects: PDGF-AA > PDGF-AB > PDGF-BB, indicating PDGF-AA as the most effective form on huc-MSCs. Therefore, PDGF-AA was selected as the representative PDGF for the formal experiments. Then, we found that not only PDGF, IGF-1 and FGF, but also TGF-β and EGF promoted the proliferation and cell cycle transition of huc-MSCs. For the first time, each of these factors was found to stimulate the migration of huc-MSCs. The effects of these factors were all significant, except that of FGF on cell cycle and migration of huc-MSCs. It might attribute to the low content of FGF in GF-PL. As shown in Table 2, we concluded that PDGF, TGF-β, IGF-1, and EGF affected all the GF-PLʹs cellular and molecular targets with significant effects respectively on 10, 8, 11, and 7 of them, while FGF affected 12 targets of GF-PL with significant effects on 5 of them. It indicates that all these factors have contributed to the effects of GF-PL in varying degrees. The content and property of each factor determine the degree of the contribution, which warrants further investigations.

**Table 3:** Known human MSC types effected by growth factors on proliferation, cell cycle transition, and migration.

Growth factors	Effected human MSC types			References
	Proliferation	Cell cycle transition	Migration	
PDGF	ASC; BMSC; DPSC; huc-MSC*; SDSC	ASC; huc-MSC*	ASC; BMSC; DPSC	^27–31^
TGF-β	ASC; BMSC; NSC	–	–	^32–34^
IGF-1	ASC; BMSC; PLSC; DPSC; huc-MSC*; NSC; PMSC	huc-MSC*	ASC	^35–41^
EGF	ASC; BMSC; HFSC; NSC;	HFSC; NSC	ASC; AMSC	^42–48^
FGF	ASC; BMSC; DPSC; DTSC; ESC; huc-MSC*; LSC; NSC; PLSC; SDSC; UBSC	huc-MSC*	BMSC; PLSC	^49–61^

“–” means undetermined.

OA is a prevalent joint disease still lacking effective therapy. Intra-articular injection of MSCs possesses efficacy of pain relief and cartilage repair, providing a therapeutic potential in OA treatment^62^. To our knowledge, little study has been conducted to demonstrate the in vivo therapeutic efficacy of huc-MSCs on knee OA. There is nonetheless a common concern on MSCs-based therapies that the survival and function of MSCs at the transplanted site are poor, often resulting in a decreased therapeutic efficacy^63^. Thus, it is necessary to improve the viability of MSCs and enhance their biological functions during the treatment. Taking advantage of the benefits of GF-PL on huc-MSCs, we combined them in this study to reach the synergistic outcome for OA treatment. For the first time, our data not only demonstrated the in vivo therapeutic activity of huc-MSCs on OA, but also revealed significant synergistic effect of combined GF-PL and huc-MSCs, according to the histopathological scoring and pain behavior (mechanical allodynia). Moreover, the combined treatment significantly reversed the OA pathological changes of anabolic biomarkers (Col2, Sox9) and catabolic biomarkers (Mmp13, Adamts4, and Adamts9), which was stronger than the single treatment of GF-PL or huc-MSCs, testifying their synergistic effect in the molecular level. Although this study revealed the adjuvant role and mechanism of GF-PL on huc-MSCs, it is not sufficient to show the synergistic relationship between them in OA treatment. Further studies are needed to explore, how GF-PL and huc-MSCs work in joint for OA cartilage repair and which mechanism is responsible for this synergism.

## Materials and methods

### Reagents and materials

Minimum essential medium-alpha modification (α-MEM) with Glutamax™-1 was purchased from Gibco BRL (NY, USA). Trypsin (0.25%) was purchased from Thermo Fisher Scientific (MA, USA). Fetal bovine serum (FBS) was purchased from CellMax (Beijing, China). Cell counting kit-8 (CCK-8) kit was purchased from Bimake (TX, USA). Cell cycle kit was purchased from BD Biosciences (CA, USA). Cell culture plates were purchased from Eppendorf (Hamburg, Germany) and Transwell chambers purchased from Corning (NY, USA). All ELISA kits were purchased from Multi Sciences (Lianke) Biotech Co., Ltd (Hangzhou, China). All human recombinant cytokines were purchased from Peprotech (London, UK). TRIzol reagent and DNase I kits were obtained from TaKaRa Biotechnology Co. Ltd. (Dalian, China). All-in-One cDNA Synthesis SuperMix kit was purchased from Biotool (TX, USA). 2× SYBR Green qPCR Master Mix (low ROX) kit was obtained from Bimake (TX, USA). The antibodies were purchased from Cell Signaling Technology Inc. (MA, USA). Monoiodoacetate (MIA) was purchased from Sigma-Aldrich (MO, USA). Human Umbilical Cord Mesenchymal Stem Cell Osteogenic Differentiation Medium kit, Human Umbilical Cord Mesenchymal Stem Cell Chondrogenic Differentiation Medium kit, and Human Umbilical Cord Mesenchymal Stem Cell Osteogenic Differentiation Medium kit were purchased from Cyagen Biosciences (Suzhou, China).

### Human PL extraction

PL was obtained using a two-step procedure consisting of double-spin centrifugation followed by freeze-thawing lysis, as described in our previous report^64^. All blood specimens used in this study were approved by the Ethical Committee of the Zhejiang Chinese Medical University. Briefly, after obtaining informed consent from healthy adult donors, whole blood was collected in tubes containing sodium citrate anticoagulant (3.2%, w/v; blood:citrate = 9:1). To obtain platelet-containing plasma separate from erythrocytes and leukocytes, each 50 ml of whole blood was centrifuged at 210×*g* for 10 min. Then the yellow plasma with buffy coat, mainly containing platelets, was collected in a new tube and centrifuged at 210×*g* for 5 min. The residual erythrocytes were discarded, before the supernatant plasma and platelet pellet were collected as platelet-rich plasma (PRP).

To measure the positive rate of platelet in our PRP, the expression of human platelet surface marker (CD41a) was analyzed as previously described^64^. Briefly, the PRP was incubated with the antibody against CD41a. After 1 h of incubation, the sample was washed with PBS and the expression of CD41a was analyzed by flow cytometer (BD FACS Calibur, BD Biosciences, CA, USA) in triplicates. Fluorescent signal intensity was recorded and analyzed by CellQuest software. The platelet number of the PRP was measured by Mindray BC-3000plus blood cell analyzer (Shenzhen, China) and standardized to 1 × 10^8^ platelets/ml, followed by repeating freeze-thaw (−80 to 37 °C) lysis for three times. The residual platelet fragments were removed by centrifugation and the obtained supernatant was collected as PL and stored at −80 °C before use.

### Immunophenotypic analysis of platelet and quantitative analysis of growth factors

The concentrations of platelet-derived growth factor (PDGF), insulin-like growth factor (IGF-1), transforming growth factor β (TGF-β), epidermal growth factor (EGF), fibroblast growth factor (FGF), and vascular endothelial growth factor (VEGF) in PL were measured in triplicates using commercially available enzyme-linked immunosorbent assay kits in accordance with each manufacturer’s instructions (Lianke Biotech Co., Hangzhou, China). The absorbance was measured using a microplate reader (Bio-Rad Laboratories, Inc., Hercules, CA, USA).

### Huc-MSCs isolation and identification

The section of human umbilical cord was obtained from the first affiliated hospital of Zhejiang Chinese Medical University with the permission of donors and was freshly processed within 4 h. The umbilical cord was cut into pieces with <2 cm^3^ volume and washed with phosphate buffered saline (PBS) (1×, pH 7.4) for three times. After removal of epidermal tissue and vessel endothelium, samples were immersed in α-MEM containing 10% FBS at 37 °C and 5% CO_2_ for incubation. The culture medium was replaced every 2 or 3 days. The isolated cells were collected as huc-MSCs and resuspended in α-MEM containing 10% FBS, with passage being carried out after confluence had occurred.

Characterization of the huc-MSCs phenotype was performed by flow cytometry. In passage 3–8, the cells were resuspended in PBS at a density of 10^6^ cells/ml and incubated with CD19, CD34, CD45, CD73, CD90, CD105, CD11b, and HLA-DR mouse antihuman antibodies, respectively. After incubation for 30 min at room temperature (RT), each cell suspension was centrifuged at 2000 rpm for 5 min. The supernatant was removed and 100 μl PBS were added to resuspend the cell pellet for flow cytometry analysis (BD FACSVerse, NJ, USA).

Three-line differentiation ability of the huc-MSCs was test by specific staining. The cells were respectively incubated in osteogenic (HUXUC-90021, Cyagen), chondrogenic (HUXUC-90041, Cyagen), and adipogenic (HUXUC-90031, Cyagen) induction medium for 14 days, according to the manufacturer’s instructions. The medium was replaced every 3 days. After confirming the morphological manifestations of differentiation, the cells were fixed with 4% polyformaldehyde for 30 min. After washing twice with PBS, osteogenic differentiation was examined with Alizarin red staining, chondrogenic differentiation was examined with Alcian blue staining, and adipogenic differentiation was examined with Oil Red O staining. All images were obtained using an inverted phase microscope (Carl Zeiss, Göttingen, Germany).

### Cell viability assay

The cell viability of huc-MSCs was determined by CCK-8 assay at 24 and 48 h. Cells were seeded on 96-well plates at a density of 4 × 10^3^ cells/well in 200 μl medium for 24 h, followed by the treatment of PL at dilution rates from 1/10 to 1/1000 and PRP at gradient concentrations corresponding to PL′s. Aliquots of each 20 μl CCK-8 solution were added to each well and incubated at 37 °C for 2 h, until the color turned to orange. The optical density (OD) value was measured at 450 nm with a microplate reader (Bio-Rad Laboratories, Inc., Hercules, CA, USA). Proliferative rate (%) = (EAA-treated OD/untreated OD) × 100. PL at dilution rates of 1/50, 1/100 and 1/200 were used as high-dose PL (PL-H), middle-dose PL (PL-M), and low-dose PL (PL-L), respectively. PRP at a concentration corresponding to PL-M was used as positive control.

### RNA sequencing analysis

Total RNA of huc-MSCs was extracted using TRIzol reagent and genomic DNA was removed using Dnase I kit. The RNA was qualitatively determined using 2100 Bioanalyzer (Agilent Technologies, CA, USA) and quantified using NanoDrop ND-2000 (NanoDrop Technologies, DE, USA). RNA purification, reverse transcription, library construction and sequencing were conducted by Shanghai Majorbio Bio-pharm Biotechnology Co., Ltd. (Shanghai, China) according to the manufacturer′s instructions (Illumina, CA, USA). RNAseq transcriptome library was prepared following TruSeqTM RNA sample preparation Kit from Illumina (CA, USA). After cDNA synthesis and quantification, paired-end RNA sequencing library was sequenced using Illumina Novaseq 6000 platform (Illumina, CA, USA). To identify differential expression genes (DEGs) between two different samples, the expression level of each transcript was calculated according to the fragments per kilobase of exon per million mapped reads (FPKM) method. Functional-enrichment analysis including GO and KEGG were performed to identify which DEGs were enriched in GO terms and KEGG pathways at *P*-value < 0.05 compared with the whole-transcriptome background.

### Cell cycle analysis

Cell cycle progression of huc-MSCs was analyzed using flow cytometry as described in our previous report^20^. Briefly, cells in the logarithmic growth phase were collected, inoculated in 6-well plates (cell density = 3 × 10^5^/well) and divided into five groups: normal control group (treated with saline), PL-L group (treated with PL-L), PL-M group (treated with PL-M), PL-H group (treated with PL-H), and PRP group (treated with PRP). After the treatment for 24 h, the huc-MSCs were harvested by centrifugation, washed with PBS, and re-suspended in cold ethanol solution (70%) at 4 °C overnight. Then, the cells were centrifuged to remove ethanol and stained with PI/RNase staining solution (10 μl of 50 μg/ml PI) at RT for 30 min. The cell cycle progression was analyzed in three replicates using flow cytometry (BD FACSVerse, NJ, USA).

### Wound healing assay

To conduct the wound healing assay, huc-MSCs in the logarithmic growth phase were inoculated in 6-well plates (3 × 10^5^/well) and artificially scratched in cross form, followed by treatment of PL with serum-free medium. The cells were observed and imaged at four different time points (0, 12, 24, and 36 h) under an inverted microscope (CarlZeiss, Göttingen, Germany). The wound area was calculated with Image J 1.47 software. Each experiment was conducted in triplicate.

### Transwell migration assay

A transwell assay was performed in 24-well transwell chambers with 8 μm nitrocellulose pore filters (Corning Costar, NY, USA) to evaluate the effect of PL on huc-MSCs migration. huc-MSCs in the logarithmic growth phase were collected, washed with PBS, and resuspended (2 × 10^5^/ml) in serum-free α-MEM medium. Aliquots of 200 µl of the huc-MSCs suspension were loaded into the upper chamber, and 600 µl serum-free α-MEM medium with PL (PL-L, PL-M or PL-H) were added into the lower chamber as PL group. The NC group applied only serum-free α-MEM medium in the lower chamber, while the PRP group applied serum-free α-MEM medium with PRP in the lower chamber. The concentration of PRP was similar to that of PL-M and can be estimated as 1% (v/v). After incubation for 18 h, the cells that passed through the membranes of the upper chambers were fixed with 4% paraformaldehyde (Servicebio, Wuhan, China) and stained with 1% crystal violet dye solution (Beyotime Biotechnology, Shanghai, China), before images of each group were captured using a microscope in different areas in three independent repeated experiments (CarlZeiss, Göttingen, Germany).

### Real time PCR

The mRNA expression of targeted genes in huc-MSCs was measured using a real time PCR (qPCR) assay on an ABI QuantStudio™ 7 Flex Real-Time PCR System (Applied Biosystems; Thermo Scientific, USA). Total RNA was extracted with TRIzol reagent and quality controlled by NanoDrop2000 spectrophotometer (Thermo Scientific, USA). cDNA reverse transcription was performed by using All-in-One cDNA Synthesis SuperMix. The PCR reaction system was 20 μl, including 10 μl SYBR Green qPCR Master Mix (low ROX), 0.4 μl PCR Forward Primer, 0.4 μl PCR Reverse Primer, 1 μl template cDNA and 8.2 μl ddH_2_O, with the following reaction conditions: initial denaturation at 95 °C for 5 min, 40 cycles of denaturation at 95 °C for 3 s, and annealing and extension at 60 °C for 30 s. β-Actin was used as reference gene and the 2^−Δ∆CT^ method was used to analyze the relative mRNA expressions (Table 4).

**Table 4:** Primer sequences used for qPCR analysis.

Gene (Human)	Forward primer	Reverse primer
*β-ACTIN*	5′-CCCGCGAGTACAACCTTCT-3′	5′-CGTCATCCATGGCGAACT-3′
*BIM1*	5′-ACCAAACCAAAGCCGTCATCA-3′	5′-GGAGCCAGTAAACGTATTGGAAG-3′
*CDC25A*	5′-TTCCTCTTTTTACACCCCAGTCA-3′	5′-TCGGTTGTCAAGGTTTGTAGTTC-3′
*CCNA2*	5′-GGATGGTAGTTTTGAGTCACCAC-3′	5′-CACGAGGATAGCTCTCATACTGT-3′
*CCNB1*	5′-AACTTTCGCCTGAGCCTATTTT-3′	5′-TTGGTCTGACTGCTTGCTCTT-3′
*CCND1*	5′-CAATGACCCCGCACGATTTC-3′	5′-CATGGAGGGCGGATTGGAA-3′
*CDK2*	5′-GCTAGCAGACTTTGGACTAGCCAG-3′	5′-AGCTCGGTACCACAGGGTCA-3′
*E2F1*	5′-CCCAACTCCCTCTACCCTTGA-3′	5′-TCTGTCTCCCTCCCTCACTTTC-3′
*E2F2*	5′-AGCTGGAACCGAGAGAACATG-3′	5′-ACACGACCAGGCGAAACC-3′
*FIBRONECTIN*	5′-AGTCCACTGAGTACCGGAGAC-3′	5′-AGGACGCTCATAAGTGTCACC-3′
*P15*	5′-AACACAGAGAAGCGGATTTC-3′	5′-AGGTCCAGTCAAGGATTTCA-3′
*P16*	5′-CATGGTGCGCAGGTTCTTG-3′	5′-CGGGATGTGAACCACGAAA-3′
*TWIST1*	5′-GTCCGCAGTCTTACGAGGAG-3′	5′-GCTTGAGGGTCTGAATCTTGCT-3′
*VEGF-A*	5′-AGGGCAGAATCATCACGAAGT-3′	5′-AGGGTCTCGATTGGATGGCA-3′
*VEGF-C*	5′-GAGGAGCAGTTACGGTCTGTG-3′	5′-TCCTTTCCTTAGCTGACACTTGT-3′
*VIMENTIN*	5′-AGTCCACTGAGTACCGGAGAC-3′	5′-CATTTCACGCATCTGGCGTTC-3′
*BECN1*-siRNA	5′-GGAAAAGAACCGCAAGATA-3′	5′-TATCTTGCGGTTCTTTTCC-3′
Gene (Rat)	Forward primer	Reverse primer
*β-actin* *Col2* *Mmp13*	5′-CCCGCGAGTACAACCTTCT-3′ 5′-CTCAAGTCGCTGAACAACCA-3′ 5′-CTATGGTCCAGGAGATGAAGAC-3′	5′-CGTCATCCATGGCGAACT-3′ 5′-GTCTCCGCTCTTCCACTCTG-3′ 5′-GTGCAGACGCCAGAAGAATCT-3′
*Adamts4*	5′-TTCGCTGAGTAGATTCGTGGAG-3′	5′-CGGACTTTTATGTGGGTTGC-3′
*Adamts9*	5′-TACAGGCAAAGGCTGGTCTC-3′	5′-CTCAGGTAGCAGGGATGGAC-3′
*Sox9*	5′-GAAAGACCACCCCGATTACA-3′	5′-TGAAGATGGCGTTAGGAGAGA-3′

### Western blot analysis

Total protein of huc-MSCs was extracted with lysis buffer (50 mM Tris-HCl, pH 7.4, 150 mM NaCl, 1 mM EDTA, 1% Triton and 0.1% SDS) containing proteinase inhibitor cocktail (Bimake, Houston, TX, USA) for 30 min on ice. The targeted protein was separated by denaturing sodium dodecyl sulfate polyacrylamide gel electrophoresis (SDS-PAGE; 6–12%) and transferred onto a nitrocellulose membrane (Sartorius Stedim, Göttingen, Germany). The membrane was blocked with 5% nonfat milk for 2 h, which was followed by overnight incubation at 4 °C with the following primary antibodies against β-actin, PCNA, Mki67, CCNB1, CCNE1, and CDK4. Following incubation with peroxidase-conjugated goat antirabbit/mouse IgG at room temperature for 2 h, each protein was visualized using Western Lightning® Plus ECL (Perkin Elmer, Inc., Waltham, MA, USA), detected using X-ray film (Kodak, Tokyo, Japan) and scanned.

### Molecular and cellular verification by small interfering RNA (siRNA) treatment

For transient knockdown of human *BECLIN1* gene, siRNA of *BECLIN1* (*BECN1*-siRNA) and nontargeting control siRNA was designed (GenePharma, Shanghai, China) to transfect huc-MSCs by using lipofectamine RNAiMAX Transfection Reagent (Thermo, Waltham, USA), according to the manufacturerʹs instruction. Western blot (WB) and cell viability assays were conducted to verify the siRNA knockdown of *BECLIN1* gene and the function of beclin1-related mechanism in the proliferative effect of PL on huc-MSCs.

### Animals and OA modeling

Male Sprague-Dawley (SD) rats weighing 180–220 g were provided by Shanghai Super B&K Laboratory Animal Co. Ltd. (Grade SPF II, SCXK (Shanghai): 2013-0016) and housed under a controlled pathogen-free condition with 12 h light/dark cycle and allowed ad libitum food and water. All rats were treated in strict accordance with the China legislation on the use and care of laboratory animals and all experiments on the rats were approved by the Medical Norms and Ethics Committee of Zhejiang Chinese Medical University (Approval number: ZSLL-2017-091). Osteoarthritis (OA) model was established applying monoiodoacetate (MIA) method as described previously^64^. Briefly, rats were intra-articularly injected with 50 μl of 30 mg/ml MIA for 7 days, followed by therapeutic treatment.

### Animal experiment

Fifty rats were randomly divided into five groups for ten in each: NC as normal control group, model as OA model group, PL as PL-M treated model group, MSC as huc-MSCs (10^6^ cells/ml) treated model group, and PL+MSC as PL-M and huc-MSCs together treated model group. Rats in the NC group were intra-articularly injected with 50 μl of saline, while all other groups were modeled. All treatments were weekly performed and lasted for 4 weeks, while the NC group was intra-articularlly given an equal volume of saline. At the end, the pain behavior parameters TWL (thermal withdrawal latency) and MWT (mechanical withdrawal threshold) were tested in triplication after the final treatment. Then all rats were sacrificed under anesthesia and their knee joints were immediately taken for histopathological analysis. The joints were resected, fixed in 10% formalin for 24 h, decalcified with 10% EDTA in PBS for 8 weeks, embedded in paraffin, sectioned into 2–3 μm thickness, and stained with SO (safranin-O). The slides were examined under microscopy and statistically graded on a scale of 0–13 by double-blind observation, according to the OARSI scoring systems^65^. The rat knee joint samples from each group were ground into powder with liquid nitrogen using a mortar and a pestle. An aliquot of the powder was treated with TRIzol reagent for total RNA extraction, and another was treated with T-PER™ Tissue Protein Extraction Reagent (Thermal Scientific) for total protein extraction. Then, qPCR and Western blot assays were conducted as described above.

### Data analysis

Data were expressed as mean values ± standard deviation (SD). Data from different groups were compared using one-way ANOVA followed by Fisher’s least significant difference (LSD) comparison. A *P*-value < 0.05 was considered to indicate a significant difference and *P*-value < 0.01 considered to indicate a very significant difference. All analyses were performed using an updated version of DPS software^66^.

## Supplementary information

Figure S1
