# Supplementary figures and images for: Growth factors-based beneficial effects of platelet lysate on umbilical cord-derived stem cells and their synergistic use in osteoarthritis treatment
Source: Cell Death Dis. 2020 Oct 14;11(10):857. doi: 10.1038/s41419-020-03045-0 (PMC7560841; doi:10.1038/s41419-020-03045-0)

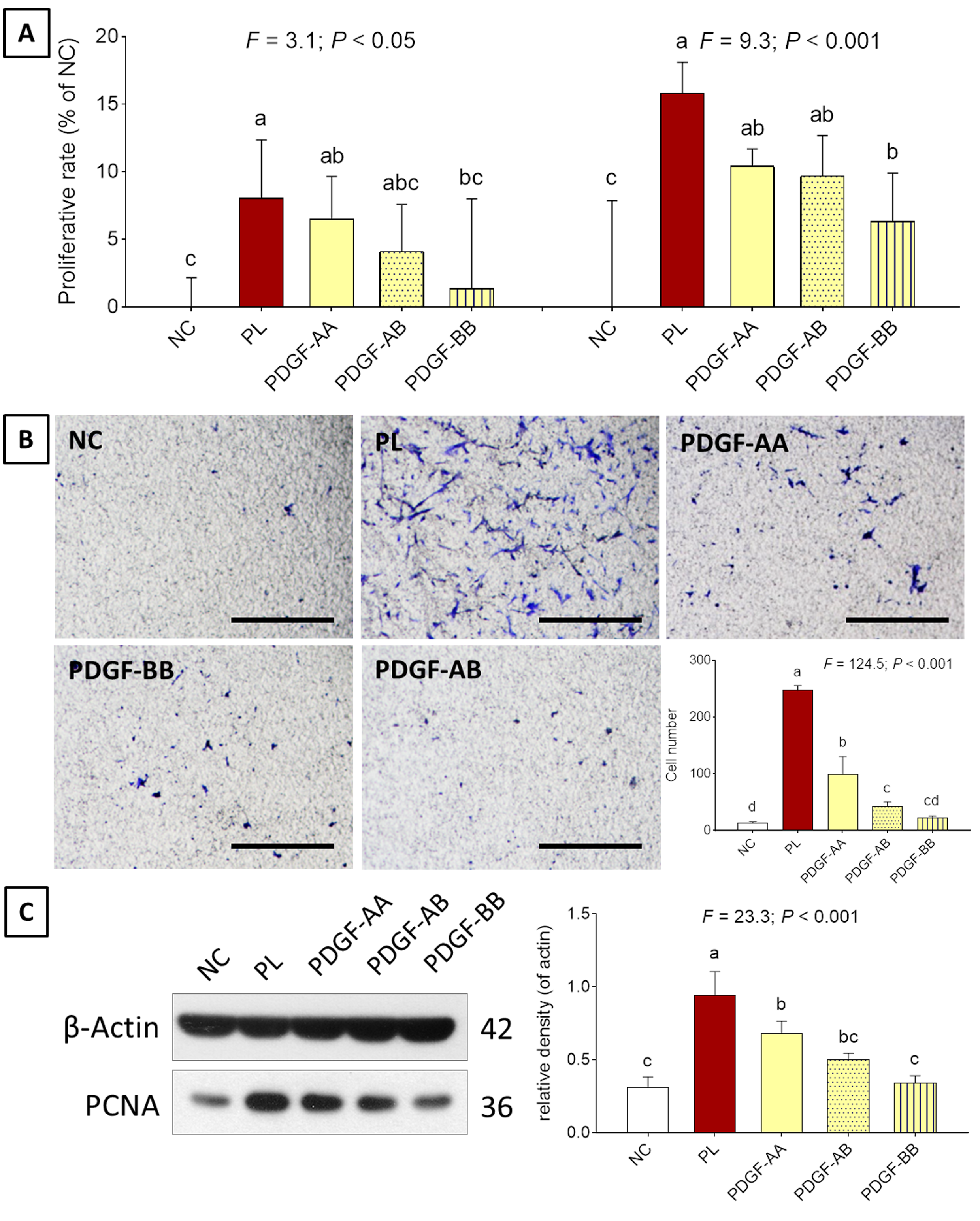

Supplement: Supplementary file 1 — Figure S1 [file 41419_2020_3045_MOESM1_ESM.png]
